# Mobile methadone dispensing in Delhi, India: implementation research

**DOI:** 10.2471/BLT.20.251983

**Published:** 2021-03-19

**Authors:** Ravindra Rao, Deepak Yadav, Roshan Bhad, Pallavi Rajhans

**Affiliations:** a National Drug Dependence Treatment Centre, Department of Psychiatry, 4th Floor, Teaching Block, All India Institute of Medical Sciences, New Delhi 110029, India.

## Abstract

**Objective:**

To assess the implementation of a mobile dispensing service to improve opioid users’ access to methadone maintenance therapy.

**Methods:**

In March 2019, we started mobile methadone dispensing in an urban underprivileged locality in Delhi, India. The doctor was available only at the main community drug treatment clinic for clinical services, while the nurse dispensed methadone from a converted ambulance. We involved patients in identifying community leaders for sensitization and in deciding the location and timings for dispensing. We conducted a retrospective chart review of the programme data collected during delivery of clinical services. We compared the numbers of patients registered for methadone therapy and their retention and adherence to therapy in the 12-month periods before and after implementation of the mobile service.

**Findings:**

The number of patients registered for therapy at the clinic increased from 167 in the year before implementation to 671 in the year after. A significantly higher proportion of patients were retained in therapy at 3, 6 and 9 months after enrolment; 9-month retention rates were 19% (32/167 patients) and 45% (44/97 patients) in the year before and after implementation, respectively. There was no significant difference in patients’ adherence to therapy between the two periods. Challenges included providing suitable dispensing hours for patients in employment and concerns of local community near to the dispensing sites.

**Conclusion:**

It is feasible to dispense methadone by a mobile team in an urban setting, with better retention rates in therapy compared with dispensing through a stationary clinic.

## Résumé

**Objectif:**

Analyser la mise en œuvre d'un service de distribution mobile destiné à améliorer l'accès des consommateurs d'opiacés au traitement de maintien à la méthadone.

**Méthodes:**

En mars 2019, nous avons commencé la distribution mobile de méthadone dans une agglomération urbaine défavorisée à Delhi, en Inde. Le médecin restait au centre communautaire de prise en charge de la toxicomanie pour assurer les services cliniques, tandis que l'infirmière circulait dans une ambulance reconvertie pour fournir de la méthadone. Nous avons demandé aux patients d'identifier les responsables au sein de la communauté, afin de les inclure dans le processus de sensibilisation et de déterminer quand et où organiser la distribution. Nous avons mené un examen rétrospectif des dossiers regroupant les données du programme, récoltées lors de la prestation des services cliniques. Enfin, nous avons comparé le nombre de patients enregistrés pour le traitement à la méthadone, leur maintien et leur adhésion au traitement sur des périodes de 12 mois, avant et après la mise en œuvre de la distribution mobile.

**Résultats:**

Le nombre de patients inscrits à la clinique en vue de recevoir un traitement est passé de 167 durant l'année précédant la mise en œuvre à 671 l'année suivante. Un pourcentage nettement plus élevé de patients ont maintenu leur traitement 3, 6 et 9 mois après l'avoir entamé; le taux de maintien après 9 mois était de 19% (32/167 patients) avant la mise en œuvre, et de 45% (44/97 patients) après celle-ci. Aucune différence notable n'a été constatée entre les deux périodes au niveau de l'adhésion des patients au traitement. Plusieurs défis se sont présentés: proposer des heures de distribution qui conviennent aux patients exerçant un emploi, et apaiser les inquiétudes de la communauté locale installée à proximité des lieux de distribution.

**Conclusion:**

Il est possible de distribuer de la méthadone par l'intermédiaire d'une équipe mobile dans un environnement urbain, et les taux de maintien du traitement sont meilleurs que ceux observés dans une clinique fixe.

## Resumen

**Objetivo:**

Evaluar la implementación de un servicio de distribución móvil para mejorar el acceso de los usuarios de opioides a la terapia de mantenimiento con metadona.

**Métodos:**

En marzo de 2019, iniciamos la distribución móvil de metadona en una localidad urbana desfavorecida de Delhi (India). El médico solo estaba disponible en la clínica principal de tratamiento de medicamentos de la comunidad para los servicios clínicos, mientras que la enfermera dispensaba la metadona desde una ambulancia transformada. Involucramos a los pacientes en la identificación de los líderes de la comunidad para la sensibilización y en la decisión del lugar y los horarios de distribución. Realizamos una revisión retrospectiva de los datos del programa recogidos durante la prestación de los servicios clínicos. Se comparó el número de pacientes registrados para la terapia con metadona y su retención y adherencia al tratamiento en períodos de 12 meses antes y después de la implementación del servicio móvil.

**Resultados:**

El número de pacientes registrados para recibir tratamiento en la clínica aumentó de 167 en el año anterior a la implementación a 671 en el año posterior. Una proporción significativamente mayor de pacientes siguió con el tratamiento a los 3, 6 y 9 meses de su inscripción; las tasas de retención a los 9 meses fueron del 19% (32/167 pacientes) y del 45% (44/97 pacientes) en el año anterior y posterior a la implantación, respectivamente. No hubo diferencias significativas en el cumplimiento terapéutico de los pacientes entre los dos períodos. Los desafíos incluyeron la provisión de un horario de distribución adecuado para los pacientes con empleo y las preocupaciones de la comunidad local cercana a los lugares de distribución.

**Conclusión:**

Es factible la distribución de metadona por un equipo móvil en un entorno urbano, con mejores tasas de retención en el tratamiento en comparación con la distribución a través de una clínica fija.

## ملخص

الغرض تقييم تنفيذ خدمة التوزيع المتنقل لتحسين حصول مستخدمي الأفيون على العلاج بصيانة الميثادون.

الطريقة في مارس/آذار 2019، بدأنا توزيع الميثادون المتنقل في منطقة حضرية محرومة من الخدمات في دلهي، الهند. كان الطبيب متواجداً فقط في العيادة المجتمعية الرئيسية للعلاج من المخدرات لتقديم الخدمات الإكلينيكية، بينما تقوم الممرضة بتوزيع الميثادون من سيارة إسعاف مجهزة. لقد أشركنا المرضى في اختيار القادة المجتمعيين للتوعية، وفي اختيار موقع وتوقيتات التوزيع. قمنا بمراجعة مخطط بأثر رجعي لبيانات البرنامج التي تم جمعها أثناء تقديم الخدمات الإكلينيكية. قمنا بمقارنة أعداد المرضى المسجلين للعلاج بالميثادون، واستمرارهم، والتزامهم بالعلاج في فترات لمدة 12 شهرًا قبل تنفيذ الخدمة المتنقلة، وبعدها.

النتائج زاد عدد المرضى المسجلين للعلاج في العيادة من 167 في العام السابق للتنفيذ، إلى 671 في العام التالي. تم الإبقاء على نسبة أعلى بشكل ملموس من المرضى في العلاج بعد 3 و6 و9 أشهر من التسجيل؛ وكانت معدلات الإبقاء لمدة 9 أشهر هي 19٪ (32/167 مريضًا)، و45٪ (44/97 مريضًا) في العام السابق للتنفيذ وبعده، على الترتيب. لم يكن هناك فرق ملموس في التزام المرضى بالعلاج بين الفترتين. تضمنت التحديات توفير ساعات توزيع مناسبة للمرضى أثناء العمل، كما تضمنت مخاوف المجتمع المحلي بالقرب من مواقع التوزيع.

الاستنتاج من المجدي توزيع الميثادون بواسطة فريق متنقل في بيئة حضرية، مع معدلات إبقاء أفضل في العلاج، مقارنة بالتوزيع من خلال عيادة ثابتة. ****

## 摘要

**目的:**

评价美沙酮流动服药车的实施情况，以提高吸毒人员美沙酮维持治疗的可及性。

**方法:**

我们于 2019 年 3 月在印度德里的一个贫困城市地区启动了美沙酮流动服药项目。医生只在主要的社区药物治疗诊所提供临床服务，而护士在改装的救护车上发放美沙酮。我们在确定社区领导（出于敏感性考虑）和确定配药地点及时间方面采纳了患者的意见，并对在提供临床服务期间收集到的项目数据进行了回顾性图表总结。我们比较了报名接受美沙酮治疗的患者数量，以及他们在流动服药车项目实施前和实施后 12 个月内的坚持用药情况。

**结果:**

在该诊所登记接受治疗的患者从实施前一年内的 167 人增加到实施后一年内的 671 人。报名后 3 个月、6 个月和 9 个月坚持治疗的患者比例明显更高；实施前和实施后 9 个月的坚持用药率分别为 19%（32/167 例）和 45%（44/97 例）。这两个阶段患者对治疗的依从性无显著差别。遇到的困难包括为在职患者提供合适的服药时间以及服药车站点附近社区居民的担忧。

**结论:**

在城市环境下，流动医疗队发放美沙酮的方案是可行的，与在固定诊所发放相比，坚持治疗的患者比例有所提高。

## Резюме

**Цель:**

Оценить эффективность внедрения мобильной службы раздачи для повышения доступности метадоновой поддерживающей терапии для потребителей опиоидов.

**Методы:**

В марте 2019 года авторы приступили к мобильной раздаче метадона в неблагополучном городском районе Дели (Индия). Врачебная помощь была доступна только в основной наркологической клинике общины, в то время как медсестра выдавала метадон из переоборудованной машины скорой помощи. Авторы вовлекали пациентов в процесс выявления активистов местной общины для проведения информационно-разъяснительной работы, а также определения места и времени раздачи метадона. Был проведен ретроспективный анализ медицинских карт данных по программе, собранных в ходе оказания медицинских услуг. Авторы сравнили количество пациентов, зарегистрированных для получения метадоновой терапии, с количеством регулярно обращавшихся за терапией пациентов на протяжении 12-месячных периодов до и после внедрения мобильной службы.

**Результаты:**

Количество пациентов, зарегистрированных для терапии в клинике, увеличилось с 167 в год до внедрения службы до 671 в год после начала работы службы. Значительно более высокая доля пациентов постоянно прибегала к терапии на протяжении 3, 6 и 9 месяцев после регистрации в программе; показатель удержания на терапии на отметке в 9 месяцев составил 19% (32/167 пациентов) и 45% (44/97 пациентов) в течение года до и года после начала работы службы соответственно. Существенной разницы в соблюдении пациентами терапии между двумя указанными периодами не наблюдалось. В число задач повышенной сложности входило обеспечение подходящих часов выдачи метадона трудоустроенным пациентам, а также решение проблем обеспокоенности местной общины вблизи мест раздачи метадона.

**Вывод:**

Мобильная раздача метадона в городских условиях оказалась целесообразной, при этом показатели удержания на терапии были выше, чем при раздаче метадона через стационарную клинику.

## Introduction

Although opioids are the second most commonly used group of illicit drugs worldwide, they cause the most harm to the health of users. Opioids account for 66% of the estimated 167 000 deaths and 80% of the 42 million years of healthy lives lost as a result of disability and premature death because of drug use disorders.^1^ Opioid overdose accounts for most drug-related deaths and is a major public health crisis in many countries.^2^^–^^4^ Use of opioids by injection is a risk factor for various health-related problems, including human immunodeficiency virus (HIV) infection and hepatitis.^5^ A systematic review in 2020 found that opioid users have substantially higher mortality than the general population. The mortality rates are even higher for those users who inject opioids and who are living with HIV.^4^

Opioid use is a major problem in India. A national survey in 2019 estimated that 22.6 million Indians aged 10–75 years had used opioids in the previous year, a prevalence of 2.1% in this age group.^6^ There are an estimated 7.7 million people who need help for their opioid use problem, and an estimated 850 000 people who inject drugs in India. Most injection drug users in India use either heroin or pharmaceutical opioids and a sizeable proportion have risky behaviours such as sharing or reuse of their injecting equipment and a history of non-fatal opioid overdose.^6^^–^^8^

Opioid agonist treatment is considered the most effective treatment for opioid dependence.^9^^–^^12^ The treatment was introduced in India in the 1990s beginning with the use of buprenorphine followed by methadone in 2012.^13^^,^^14^ Presently, there are more than 250 opioid agonist treatment centres dispensing buprenorphine and more than 17 methadone maintenance treatment centres supported by various government agencies in India. Despite this expansion, there is limited coverage of opioid agonist treatment; only around 25 000–30 000 individuals dependent on opioids receive opioid agonist treatment in India.^13^ In most opioid agonist treatment centres, the client is required to visit the centres daily to receive their medicine, which may affect their retention in treatment. While some centres allow patients to take buprenorphine home, methadone is not provided as take-home medicine in India. Thus, improvement in the coverage of opioid agonist treatment, and the devising of newer strategies to reach out to a larger number of individuals with opioid dependence, are needed. 

Delhi, with a population of around 19 million, is home to about 370 000 people with opioid use disorder and an estimated 88 000 people who inject drugs.^6^ There are 12 opioid agonist treatment clinics in the area, which provide clinic-based buprenorphine dispensing on a daily basis. Some areas of the city have a large concentration of individuals with opioid use disorder. Our methadone maintenance treatment clinic initially attracted people living near to the clinic, but increasingly started enrolling patients living further from the clinic. Over time, however, these patients reported difficulties with visiting the clinic daily for their methadone dose, especially after completing 2–3 months of treatment, when they had quit illicit opioids and wanted to focus on their work. These problems were reflected in the low retention rates of patients coming from more distant areas. To address this challenge, we planned to dispense methadone in areas further from the clinic using a converted ambulance. Dispensing of methadone through mobile vans has been tried in other parts of the world.^15^^,^^16^ We report an initial assessment of the implementation of the mobile dispensing service. We discuss the dispensing model, its achievements and the challenges to its implementation.

## Methods

### Setting

The community drug treatment clinic run by the authors’ institution is the only centre providing methadone in Delhi. The clinic is located in an urban area with a sizeable number of people using illicit opioids. The clinic started providing methadone from 2012 for people staying in a catchment area of around 6–8 km. The community clinic is housed in a government building and uses local resources for providing other health-care services to the methadone maintenance treatment clients (such as testing or treatment for HIV and tuberculosis). The team of three to four doctors and a counsellor is available twice per week, while three nurses are available on all days to dispense methadone. By 2016, the clinic was dispensing methadone to about 150 patients each day.

### Implementation

We planned a mobile service that would only dispense methadone for patients who were assessed and followed up (including changes in the methadone dose, if needed) in the existing community drug treatment clinic. We held meetings with the clinic’s patients to discuss the plan and to finalize the dispensing locations of the ambulance and the timings of dispensing. An ambulance was procured, and converted to be suitable for dispensing methadone. Before beginning the service, we informed the local community leaders identified by the patients through one-to-one meetings. The police, traffic authorities and local health authorities were also informed, and formal permissions were taken from them. At these meetings we emphasized the medical model of addiction and the role of medicines in treatment of opioid dependence. We also invited local stakeholders to the inauguration programme and sought their support for the mobile service. 

We began dispensing methadone through the mobile service from 22 March 2019. Initially, we chose one dispensing location close to a junction of busy streets, 4–5 km distance from the clinic. The location was chosen so that most patients would find it easy to receive their methadone dose in the morning from 08:30–11:00 and then go to their work. Within 3–4 months, the mobile van was dispensing methadone daily to about 100 patients registered at the clinic for treatment. Encouraged by the response, we started dispensing methadone at 11:30–13:30 from another location 4–5 km from the clinic while still dispensing from the first station at 08:30–11:00. We started dispensing methadone at the second site from 11 October 2019, and by March 2020 close to 80 patients received their methadone daily from the second site. About 400 patients received methadone on any given day through the three dispensing sites (from the clinic and from the ambulance at two dispensing sites).

A nurse, a helper and a guard, besides the driver of the ambulance, ran the mobile service. The staff collected the methadone supplies from the community drug treatment clinic daily in the morning before beginning dispensing and returned unused treatments to the clinic after completing dispensing. We have digitized the dispensing records, so patients are free to consume their doses from any of the three dispensing points on any day. Patients are followed up with a doctor and counsellor in the main clinic about every 4–6 weeks when their methadone prescription is due for renewal. While dispensing methadone, a nurse interacts briefly with the patients and refers them to a doctor in the clinic if they have any medical problems. Patients are also referred to the doctor for titrating their methadone dose if they do not take methadone for more than 5 consecutive days.

We use the following criteria for selecting patients for registration and initiation of methadone maintenance treatment at the clinic: diagnosis of opioid dependence syndrome (diagnosed as per the International Statistical Classification of Diseases and Related Health Problems, 10th revision, criteria); residing in the catchment area of the clinic (of about 6–8 km); suitable for opioid agonist treatment as decided by the doctor (based on the duration of opioid use and complications due to opioid use); and willing to follow the clinic protocols (regular follow-up with the doctor, coming to the clinic or ambulance daily to receive methadone). The clinic provides methadone maintenance treatment with no restrictions on age or sex of patients.

### Data collection

To assess the implementation of the mobile service we carried out a retrospective review of the records maintained in the clinic. We used clinical data collected routinely at baseline and at follow-up to assess changes in patient outcomes after introducing mobile dispensing. The output variables of interest were enrolment in methadone maintenance treatment, retention in treatment and adherence to treatment. We compared data from two time periods: 12 months before the start of mobile methadone dispensing (March 2018 to February 2019) and 12 months following implementation (March 2019 to February 2020). We included all patients started on methadone from our clinic during the study periods. 

The doctors maintained the clinical records, while the nurses maintained the medicine dispensing records. Just before the start of the mobile service, we moved the clinic and dispensing records to a locally developed online system so that synchronized dispensing records could be maintained at different dispensing points. This meant that the clinical and dispensing records for 2018–2019 were available on paper, while the digital records for 2019–2020 were available online. One of the authors collected relevant data from the records of each patient and entered the data into an Excel spreadsheet (Microsoft Corp., Redmond, United States of America, USA) prepared for the study. 

We conducted the retrospective chart review after receiving ethical clearance from our institution.

### Data analysis

We calculated the retention rates of each patient over the third, sixth and ninth months after starting treatment. We considered those patients who received methadone in the month considered for analysis as retained for that month. For example, we considered a patient as retained for 3 months if he or she received methadone (even once) in the third month after enrolment. We counted patients who registered at least 3 months before February 2020 in the denominator for calculating 3-month retention. Similarly, those patients registered at least 6 months before February 2020 were counted in the denominator for calculating 6-month retention, and so on. We calculated the mean of the adherence rates (that is, the proportion of days for which the patient took the prescribed doses) of those retained for the third, sixth and ninth month.

We calculated descriptive statistics: the mean (standard deviation, SD) or median (interquartile range, IQR) for continuous variables, and percentages for categorical variables. We compared the data for patients enrolled in the year preceding the intervention and those enrolled in the following year using χ^2^ tests for the categorical variables and independent sample *t*-tests for the continuous variables. We used Levene’s test to measure equality of variance for the continuous variables. We performed the statistical analysis using IBM® SPSS® Statistics, version 25.0 (IBM, Armonk, USA). A two-sided *P* value < 0.05 was considered statistically significant.

## Results

### Patients’ characteristics

The analysis included 838 patients: 834 men (99%) and four (1%) women. The age range of patients was 14–48 years with a mean of 24.5 years (SD: 5.4). Most patients (568; 68%) were unmarried, while 267 patients (32%) were married and three patients were divorced. A total of 511 patients (61%) were employed at the time of enrolment, while 312 (37%) were unemployed (data were unavailable for 15 patients). Most patients had received some form of education; only 65 (8%) were uneducated. The median monthly family income was 20 000 Indian rupees (IQR: 10 000–30 000; 275 United States dollars, US$; IQR: 138–414).

Within their lifetime, almost all patients (796; 95%) had used tobacco; 473 (56%) had used alcohol and 622 (74%) had smoked cannabis. The proportion of patients with current (past 1 month) use of tobacco, alcohol and cannabis were 95% (793 patients), 31% (262 patients) and 65% (545 patients), respectively. Patients with lifetime dependence on tobacco, alcohol and cannabis comprised 90% (751 patients), 3% (26 patients) and 29% (242 patients) of the total, respectively. The mean age of onset of illicit opioid use was 19.7 years (SD: 5) and the median duration of opioid dependence syndrome was 4 years (IQR: 2–6). Most patients (717; 86%) had used opioids by inhalation, while 121 (14%) patients had ever injected opioids in their lifetime; 29 patients (3%) had shared their injecting equipment at least once. A total of 51 patients (6%) reported getting tested for HIV, of whom six patients reported being HIV-positive. A total of 192 participants (23%) admitted to carrying out illegal activities and 11% (88 patients) had ever been imprisoned.

### Retention and adherence 

Of the total patients studied, 167 patients (20%) were enrolled in methadone therapy in the year before implementation of the mobile service and 671 patients (80%) in the following year. Out of the 668 patients enrolled at least 3 months before the end of the observation period, 402 (60%) were retained on methadone maintenance treatment (Table 1). Similarly, 207 (46%) of the 447 patients enrolled at least 6 months before the end of the observation period were retained. Of the 262 patients enrolled 9 months before the end of the observation period, 76 (29%) were retained. In the 12-month period after the start of the service, a significantly higher proportion of patients were retained in the third month (*P* < 0.01), sixth month (*P* < 0.01) and ninth month (*P* < 0.01) compared with the 12-month period before implementation.

**Table 1:** Retention in therapy in the year before (March 2018 to February 2019) and year after (March 2019 to February 2020) implementation of mobile methadone dispensing by a clinic in Delhi, India

Length of observation period	Total (*n* = 838)			Before implementation (*n* = 167)			After implementation (*n* = 671)			Before and after comparison	
	No. of patients observed	No. (%) of patients retained in therapy		No. of patients observed^a^	No. (%) of patients retained in therapy		No. of patients observed	No. (%) of patients retained in therapy		*χ^2^* value (df)	*P*
3 months	668	402 (60)		167	84 (50)		501	318 (63)		9.071 (1)	< 0.01
6 months	447	207 (46)		167	60 (36)		280	147 (53)		11.554 (1)	< 0.01
9 months	262	76 (29)		167	32 (19)		97	44 (45)		21.105 (1)	< 0.01

df: degrees of freedom.

^a^ As all patients enrolled before implementation were enrolled at least 1 year before the data analysis, the number of patients observed at 3, 6 and 9 months remain the same.

Notes: *n* is the total number of patients enrolled. Retention is the number of patients retained in therapy in the third, sixth and ninth months after enrolment as a proportion of the number of patients enrolled in methadone maintenance therapy at the clinic.

The mean proportions of patients adhering to therapy of those retained at the third, sixth and ninth month were compared using independent sample-*t*-test (Table 2). Levene’s test for equality of variance was fulfilled for all the three time periods considered. There was no significant difference between the adherence rates in the year before and after the start of mobile dispensing. 

**Table 2:** Adherence to therapy in the year before (March 2018 to February 2019) and year after (March 2019 to February 2020) implementation of mobile methadone dispensing by a clinic in Delhi, India

Length of observation period	Before implementation (*n* = 167)			After implementation (*n* = 671)			Before and after comparison	
	No. of patients retained in therapy	Mean (SD) % adherence		No. of patients retained in therapy	Mean (SD) % adherence		*t-*value (df)	*P*
3 months	84	61.6 (25.4)		318	58.2 (22.3)		1.188 (399)	0.24
6 months	60	66.1 (23.2)		147	63.7 (24.8)		0.647 (204)	0.52
9 months	32	58.9 (25.6)		44	61.3 (27.2)		0.370 (69)	0.71

df: degrees of freedom; SD: standard deviation.

Notes: *n* is the total number of patients enrolled. We recorded the number of days each patient took the prescribed methadone dose and calculated the proportion of days on which each patient adhered. Mean adherence is the average percentage adherence among patients retained in the third, sixth and ninth months after enrolment.

## Discussion

The few existing stationary opioid agonist treatment clinics in Delhi would not be enough to meet the needs of a large population with opioid use disorder. The mobile methadone dispensing scheme provides an alternative model to the existing clinic-based dispensing of methadone and has the potential to reach a large number of patients. Our results show that there was an increase in the uptake of methadone therapy as well as significant improvement in retention rates after introduction of the mobile service. The overall experience thus far shows the utility of the model in expanding access to methadone for opioid dependence.

Studies have shown that strategies that improve users’ access to methadone by increasing access points, such as pharmacy-based dispensing, lead to improvements in treatment outcomes.^17^^,^^18^ In India, the existing narcotic laws do not allow pharmacies to dispense methadone. Therefore, direct dispensing from vans in densely populated urban locations can be an alternative for increasing treatment access and retention rates, as seen in our study. The mobile service uses the hub-and-spoke model. The doctor is available only at the hub (the main clinic) twice a week for clinical assessment, starting patients on methadone, titrating methadone doses on follow-up, and referral to other health-care providers as necessary. The nurse is available at different spokes (dispensing locations of the van) at different times of the day. In India, the nurses dispense methadone in most opioid agonist treatment centres rather than pharmacists. The current model allows for optimal utilization of human resources in providing methadone services by nurses and can be a cost-effective model for helping large populations of patients with opioid dependence.

Another important feature of this initiative has been the involvement of patients in the programme right from the beginning in identifying community leaders who needed to be consulted and in deciding the locations and timings of mobile dispensing. Community participation in programmes improves treatment outcome in substance use disorders.^19^^–^^21^ Despite easing access, the retention rates of patients on methadone in our study are lower compared with other studies.^11^^,^^22^^,^^23^ Further studies are needed to find out the reason for lower retention rates.

The programme incurred only a modest cost of running the van, which was funded by the Ministry of Health and Family Welfare of India. The ambulance was purchased for 1.5 million Indian rupees (about US$ 21 000). The recurring cost for running the mobile van (fuel cost and human resource) is about 100 000 Indian rupees (US$ 1500) per month. This translates to about US$ 7–8 per patient per month. 

The advantages of mobile methadone dispensing were greater in the pandemic coronavirus disease 2019 (COVID-19). India imposed a strict lockdown in March 2020 to curb the pandemic.^24^ During the lockdown there were severe restrictions in accessing what were seen as non-essential health-care services, including addiction treatment services.^25^ People with substance use disorders had difficulty travelling to addiction treatment facilities for their medication because of restrictions on the movement of people and vehicles, including suspension of government transport. Our mobile service continued dispensing methadone even during the lockdown. As most of our patients did not have to travel far for their dose, travel restrictions did not affect their treatment. Some users reported police harassment during the lockdown when they came out of their house for their dose. The clinic decided to allow take-home methadone doses for up to 3–7 days for each patient (depending on the methadone dose of the patient). We did this to reduce crowding near the van and to reduce possible exposure of methadone users to COVID-19 due to daily travel. The presence of mobile dispensing in their locality helped the users to access their medicine even during the COVID-19 pandemic.

Implementing a mobile service was not without challenges. The first challenge we faced was the dispensing times. Many patients preferred to access the service in the morning, as it allowed them to reach their workplace after their methadone dose. However, we did not have the capacity to provide only early morning services. To resolve this issue, we asked users of the second dispensing location who were working in regular offices to take their medicine from the main clinic which began dispensing from 08:00. Many others who were working in small factories in the vicinity were asked to take their methadone from 11:30–13:30 at the second location during their tea break. Soon after starting dispensing from the second site, some trustees of a nearby place of worship demanded that the dispensing point be shifted. They feared that people would come to receive their methadone in an intoxicated state and create a nuisance for worshippers. They were also apprehensive about potential thefts in the building. They persisted with their demand despite assurances that these apprehensions were unfounded. Because of their pressure, we shifted the ambulance 500 m away from the temple. The area where the second dispensing location is based also witnessed communal riots in February 2020, and we suspended methadone dispensing from the second location for a couple of days. The patients were requested to take their methadone dose from the main clinic for a few days until the end of the riots.

Despite these challenges, the mobile service has been able to attract new patients to methadone maintenance and has improved retention on methadone. As the mobile methadone van is part of the overall addiction treatment system of our institution, we do not anticipate halting the service in the future. We hope that the benefits of this delivery system will prompt the health authorities of Delhi to scale up these services to other parts of the city.

In conclusion, dispensing of methadone through mobile vans can help in providing methadone maintenance treatment to large numbers of patients with opioid dependence compared with stationary clinic-based dispensing of methadone. This approach can also help in improving opioid users’ retention on methadone.
